# Determinants of complete antenatal care in Sub-Saharan Africa: a multilevel analysis of Demographic and Health Surveys from 28 countries

**DOI:** 10.3389/fgwh.2026.1845317

**Published:** 2026-09-23

**Authors:** Hamse Adam Abdi, Osman Mohmed Osman, Abdulkadir Mohamed Nuh, Hamse Arab Ali, Abdisalam Hassan Muse

**Affiliations:** 1 College of Medicine and Health Science, University of Hargeisa, Hargeisa, Somaliland; 2 HIR Institute for Research and Development, Hargeisa, Somaliland; 3 Faculty of Science and Humanities, School of Postgraduate Studies and Research (SPGSR), Amoud University, Borama, Somaliland

**Keywords:** 28 countries, complete ANC quality, Demographic and Health Survey (DHS), determinants, Sub-Saharan Africa

## Abstract

**Background:**

Antenatal care (ANC) quality is assessed by the number of recommended ANC services a woman receives during pregnancy, including blood pressure checks, urine and blood sampling, and administration of iron supplements. The World Health Organization recommends that, to make ANC services an effective preventive measure, the content and quality of care should be routinely monitored.

**Methods:**

A total of 394,067 women who gave birth 5 years before the survey were included. We used Stata version 17 for data management and analysis. A multilevel logistic regression model was fitted. Finally, the adjusted odds ratios (aOR) with 95% confidence intervals (CIs) are reported. Statistical significance was defined as *P*-value < 0.05.

**Results:**

Complete ANC was significantly higher among women with primary (aOR 1.63, 95% CI 1.59–1.66), secondary (aOR 2.31, 95% CI 2.26–2.37), or higher education (aOR 2.99, 95% CI 2.84–3.15); among those living in poorer (aOR 1.05, 95% CI 1.03–1.08), richer (aOR 1.04, 95% CI 1.01–1.07), or the richest households (aOR 1.14, 95% CI 1.10–1.18), and among women who wanted later pregnancies (aOR 1.25, 95% CI 1.23–1.28). It was lower among women with unwanted pregnancies (aOR 0.54, 95% CI 0.53–0.56), those who were working (aOR 0.90, 95% CI 0.89–0.92), those exposed to media (aOR 0.97, 95% CI 0.95–0.99), those living in female-headed households (aOR 0.96, 95% CI 0.94–0.98), and among all ever-married women (married aOR 0.67, 95% CI 0.63–0.71; cohabiting aOR 0.68, 95% CI 0.64–0.72; widowed aOR 0.38, 95% CI 0.33–0.43; divorced aOR 0.66, 95% CI 0.60–0.74; separated aOR 0.63, 95% CI 0.57–0.68).

**Conclusion:**

This study demonstrates that fewer than 2 in 10 women in Sub-Saharan Africa receive all recommended antenatal care services, indicating low coverage. The key determinants of complete antenatal care were identified, including household wealth, maternal education, pregnancy intention, marital status, media exposure, sex of the household head, and maternal employment status.

## Background

Antenatal care (ANC) is a critical component of maternal health services, providing opportunities for preventive interventions that improve maternal and neonatal outcomes. ANC visits enable healthcare providers to deliver essential services such as health education, clinical screening, and nutritional supplementation, which are vital for ensuring safe pregnancy and childbirth (1). Antenatal care visits are an ideal time to advise mothers and families on essential pregnancy care to reduce stillbirths and neonatal deaths and to develop a birth preparedness plan; thus, the quality of antenatal care is typically measured by the receipt of essential recommended services during pregnancy, including blood pressure checks, urine and blood tests, and iron supplementation (2–4).

The World Health Organization (WHO) advises that pregnant women should engage in comprehensive antenatal care, involving a minimum of eight contacts throughout pregnancy, to enhance maternal and fetal outcomes. It is crucial to monitor both the content and quality of antenatal care services to ensure the consistent delivery of recommended interventions (5). Complications arising during pregnancy are recognized as a primary cause of maternal mortality, stillbirth, and neonatal mortality, with inadequate quality of ANC being a contributing factor (6). Insufficient utilization of antenatal care services diminishes the opportunities to provide all recommended components of quality ANC.

Antenatal care is instrumental in the early detection and management of pregnancy-related complications and ensures access to essential preventive services, such as vaccinations, diagnostic testing, and health education. Women who receive high-quality antenatal care are more likely to utilize skilled delivery services and emergency obstetric care, thereby reducing the risk of maternal and neonatal morbidity and mortality (7–12). Early pregnancy, particularly among adolescents, represents a significant global public health issue affecting both developed and developing nations (13). The provision of care for mothers and infants before and during birth is vital. Although some debate the efficacy of prenatal care in preventing serious maternal health issues, evidence indicates that it can avert complications for both mothers and infants through early intervention or appropriate management (14).

Research indicates that unplanned pregnancies frequently receive insufficient care, while maternal mortality can be averted through the use of high-quality ANC services (8–10). High-quality ANC services facilitate the early identification and management of complications, thereby enhancing the likelihood of a safe delivery (7, 8, 15).

Reducing maternal mortality continues to be a global priority, as outlined in Sustainable Development Goal 3 (SDG 3). Despite significant advancements, approximately 800 women die daily from pregnancy-related causes worldwide. Sub-Saharan Africa (SSA) experiences a disproportionate burden, contributing to nearly two-thirds of global maternal deaths (9–12).

Research conducted across various sub-Saharan African nations has identified socioeconomic and demographic factors, including education, household wealth, marital status, media exposure, and place of residence, as significant determinants of ANC quality (16). Substantial evidence suggests that antenatal care services in Sub-Saharan Africa frequently do not adhere to recommended quality standards (17). The majority of existing studies concentrate on service utilization or facility readiness, rather than on the quality and comprehensiveness of the ANC services received by women. Furthermore, numerous studies are confined to single-country analyses or small regional samples, which limits the generalizability of their findings. There is a paucity of research examining the influence of reproductive factors, such as age at first birth, on the quality of antenatal care. Even fewer studies have employed multilevel analytical approaches that consider the hierarchical structure of Demographic and Health Survey (DHS) data. As a result, there is limited multi-country evidence on the determinants of comprehensive antenatal care across Sub-Saharan Africa (13, 14).

This study aims to identify the determinants of complete antenatal care among women in Sub-Saharan Africa using nationally representative Demographic and Health Survey data from 28 countries. By applying multilevel modeling techniques, the study examines both individual- and community-level factors influencing ANC quality. The findings are expected to inform evidence-based policies and interventions aimed at improving maternal healthcare services and supporting progress toward Sustainable Development Goal 3 in the region.

## Method and material

### Study area

The sub-Saharan region includes all African countries and areas located fully or partly south of the Sahara Desert. Over 500 million women live in this region, and nearly half of them are between 15 and 49 years old (18). This study was conducted across 28 Sub-Saharan African countries, representing Western, Central, Eastern, and Southern Africa and reflecting diverse geographical, socioeconomic, and maternal healthcare contexts.

### Data source

The DHS cross-sectional surveys collected information from women aged 15 to 49 about health and related topics. Permission to use the DHS data was obtained through the official DHS website. The surveys used a two-stage sampling method. First, each country was divided into groups. Enumeration areas were selected for each group, and lists of households in these areas were made. In the second stage, households from these lists were chosen. Women aged 15–49 in these households were interviewed in person by fieldworkers.

### Sample size

The study used data from 394,067 women. These data came from the DHS and were cleaned and prepared. The participants were from 28 countries in Sub-Saharan Africa (Table 1).

**Table 1:** Sample size per country.

Country	DHS survey year	Sample size
Angola	2023–2024	2,841
Burkina Faso	2021	7,258
Benin	2017–2018	8,416
Burundi	2016–2017	7,648
DR Congo	2023–2024	16,412
Côte d’Ivoire	2021–2022	5,890
Gabon	2012	3,302
Ghana	2022	4,844
Gambia	2019–2020	5,320
Guinea	2018	4,412
Kenya	2022	4,754
Comoros	2012	1,772
Lesotho	2023	1,164
Madagascar	2018–2019	16,146
Mauritania	2015–2016	18,414
Malawi	2015–2016	20,628
Mozambique	2022–2023	12,336
Nigeria	2018	76,086
Niger	2012	31,962
Namibia	2013	5,826
Rwanda	2019–2020	9,492
Sierra Leone	2019	13,356
Senegal	2023	14,850
Chad	2014–2015	17,022
Togo	2013–2014	12,468
Tanzania	2022	17,238
Uganda	2016	29,658
South Africa	2016	2,346
Zambia	2018	15,816
Zimbabwe	2015	6,390
		**394,067**

The bold value represents the total sample size across all 28 countries (N = 394,067).

### Study variables

#### Outcome variable

The outcome variable in this study was the quality of ANC, categorized as “complete” or “incomplete.” ANC was considered complete if a respondent received all of the following four essential services: (1) measurement of blood pressure, (2) collection of urine and (3) blood samples, and (4) provision of iron supplements. Respondents who received fewer than all four services were classified as receiving incomplete ANC.

After harmonizing and recoding variables across the country datasets, the analysis was restricted to women aged 15–49 years who had a birth in the preceding 5 years and complete information on the selected ANC components. Those with incomplete observations were excluded using complete-case analysis, and no imputation was performed. The independent variables in this study include residence, education level, wealth index, current pregnancy wanted, marital status, employment status, media exposure, age of the respondent, and sex of household head. Blood pressure measurement, urine testing, blood testing, and iron supplementation were selected because they are essential ANC interventions consistently available across the included DHS datasets and have been used in previous multi-country DHS studies to operationalize ANC quality; however, this measure represents the complete receipt of the selected ANC components rather than all dimensions of ANC quality.

### Data analysis

Stata version 17 was used for the analysis. Descriptive statistics were conducted using cross-tabulation and calculating frequencies and percentages to show how individual- and community-level characteristics were distributed (Table 2). The chi-squared test was used to assess whether individual- and community-level factors correlated with ANC quality. In the multivariable, multilevel analysis, statistical significance was defined as *p*-value <0.05. Cases with missing data on the outcome or explanatory variables were excluded using complete-case analysis, and no statistical imputation was performed. For the data to be representative and to account for the probability of unequal selection between clusters, both descriptive and analytic analyses were carried out after weighting the data using individual weights for women (v005). We then generated individual weights by dividing by 100,000. As the DHS data are hierarchical, meaning there are some variables nestled within other variables, and to examine cluster variability, we created the following four models: model 0, the null model, with only the dependent variable; model 1, including the dependent variable and individual-level variables; model 2, including the dependent variable and community-level variables; and model 3, including the dependent variable and individual- and community-level variables. To assess clustering and check the appropriateness of multilevel logistic regression, we calculated the intraclass correlation coefficient (ICC), Akaike information criterion (AIC), Bayesian information criterion (BIC), and likelihood ratio (LR). The ICC of the null model showed an ICC of 28.46%, a log-likelihood of −15,074.27, an AIC of 30,152.54, a BIC of 30,173.87, and LR of *χ*^2^ = 964.78, *p* < 0.001.

**Table 2:** Univariate analysis of determinants of quality of ANC among women in Sub-Saharan Africa.

Variable	Category	Frequency	Percentage
Place of residence	Urban	96,534	24.50
	Rural	297,533	75.50
Highest educational level	No education	184,821	46.90
	Primary	130,078	33.01
	Secondary	70,855	17.98
	Higher	8,313	2.11
Sex of household head	Male	334,062	84.77
	Female	60,005	15.23
Wealth index	Poorest	110,312	27.99
	Poorer	92,103	23.37
	Middle	82,160	20.85
	Richer	64,029	16.25
	Richest	45,463	11.54
Media exposure	Yes (1)	296,718	75.30
	No (2)	97,349	24.70
Age group	15–19	6,490	1.65
	20–24	32,620	8.28
	25–29	61,780	15.68
	30–34	76,234	19.35
	35–39	83,960	21.31
	40–44	70,725	17.95
	45–49	62,258	15.80

## Results

The prevalence of complete ANC was 13% in Angola, 10% in Burkina Faso, 24% in Benin, 25% in Burundi, 12% in Democratic Republic of the Congo, 13% in Côte d’Ivoire, 26% in Ghana, 16% in Gambia, 12% in Guinea, 15% in Kenya, 18% in Comoros, 33% in Lesotho, 17% in Madagascar, 10% in Mauritania, 16% in Malawi, 19% in Mozambique, 10% in Nigeria, 7% in Niger, 22% in Namibia, 28% in Rwanda, and 19% in Sierra Leone (Figure 1). Overall, the findings indicate substantial variation in ANC quality across the countries, with Lesotho recording the highest percentage and Niger the lowest, suggesting disparities in maternal healthcare access, service quality, and utilization of antenatal care across African countries.

**Figure 1:**
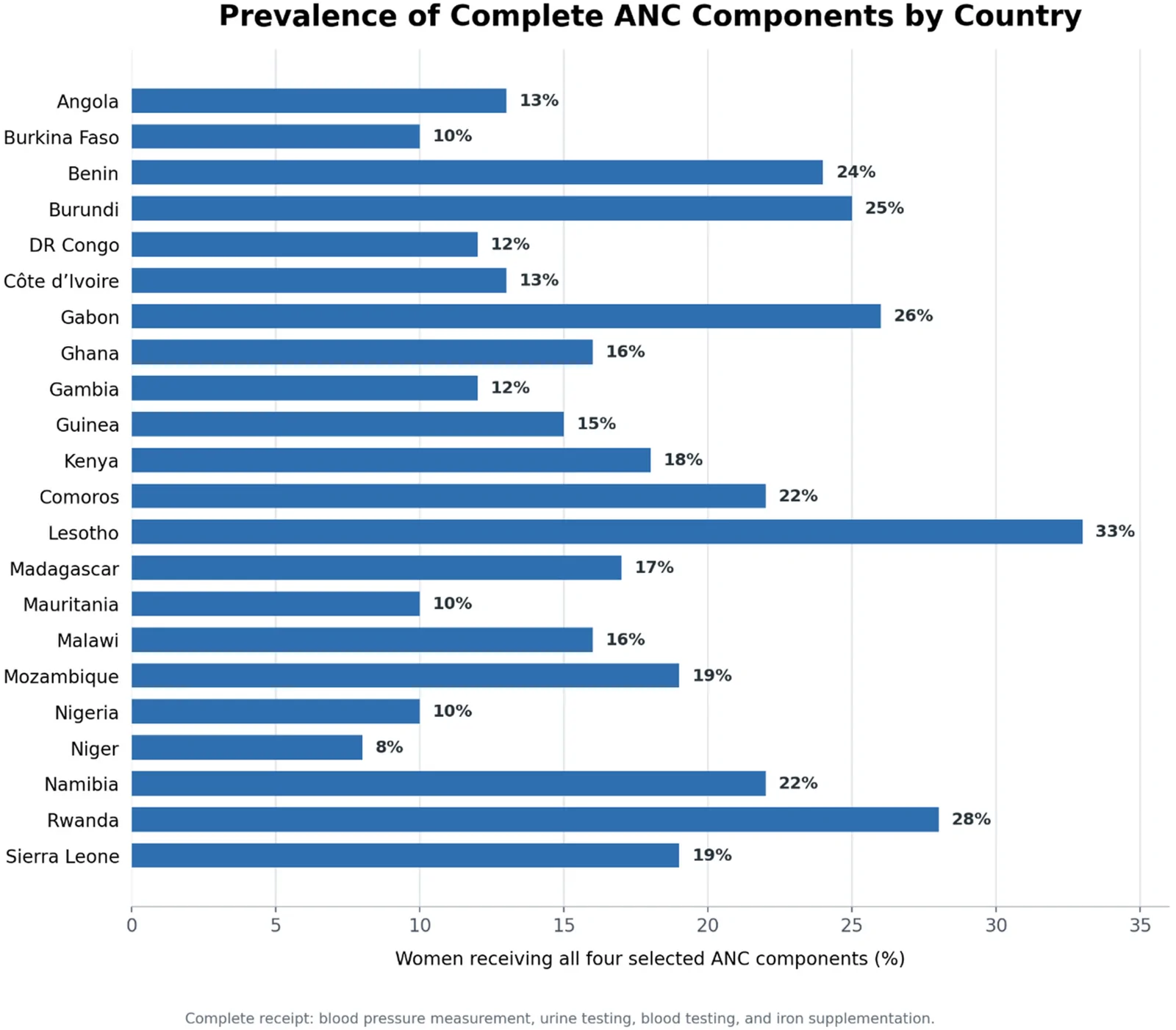
Prevalence of complete ANC in SSA.

### Determinants of quality of antenatal care in Sub-Saharan Africa: DHS from 28 countries

In model 3, maternal education, wealth, pregnancy intention, employment status, media exposure, marital status, and sex of household head were significant predictors of receiving complete ANC (Table 3). Compared with women with no education, women with higher educational attainment had significantly greater odds of receiving complete ANC. Women with primary education were 63% more likely to receive complete ANC (aOR = 1.63, 95% CI: 1.59–1.66, *p* < 0.001). Similarly, women with secondary education had 131% higher odds of receiving complete ANC (aOR = 2.31, 95% CI: 2.26–2.37, *p* < 0.001), while women with higher education were nearly three times more likely to receive complete ANC compared to women with no education (aOR = 2.99, 95% CI: 2.84–3.15, *p* < 0.001). Regarding wealth, women living in the poorer (aOR = 1.05, 95% CI:1.03–1.08), richer (aOR = 1.04, 95% CI:1.01–1.07), and richest households (aOR = 1.14, 95% CI:1.10–1.18) were more likely to receive complete ANC compared with those living in the poorest households. Regarding pregnancy intention, those who wanted a later pregnancy (aOR = 1.25, 95% CI: 1.23–1.28) and those who had an unwanted pregnancy (aOR = 0.54, 95% CI: 0.53–0.56) were more and less likely to receive complete ANC, respectively, compared to those with a wanted pregnancy. Working women had an aOR of 0.90 (95% CI: 0.89–0.92) for receiving complete ANC vs. those who did not work, and media-exposed women had an aOR of 0.97 (95% CI: 0.95–0.99) vs. those who had no exposure to media. Regarding marital status, those who were married (aOR = 0.67, 95% CI: 0.63–0.71), living with a partner (aOR = 0.68, 95% CI: 0.64–0.72), widowed (aOR = 0.38, 0.33–0.43), divorced (aOR = 0.66, 95% CI: 0.60–0.74), or separated (aOR = 0.63, 95% CI: 0.57–0.68) were less likely to receive complete ANC vs. those who were never married. Finally, women in female-headed households had an aOR of 0.96 (95% CI: 0.94–0.98) for receiving complete ANC compared with male-headed households.

**Table 3:** A multilevel analysis of determinants of quality of antenatal care in Sub-Saharan Africa: DHS of 28 countries.

Variable	Category	*p*-value	Model 3 aOR (95% CI)	*p*-value
Women’s education (v106)	No education (Ref)	–	1.00	–
	Primary	–	1.63 (1.59–1.66)	<0.001
	Secondary	–	2.31 (2.26–2.37)	<0.001
	Higher	–	2.99 (2.84–3.15)	<0.001
Sex of household head (v151)	Male (Ref)	–	1.00	–
	Female	–	0.96 (0.94–0.98)	0.001
Wealth index (v190)	Poorest (Ref)	–	1.00	–
	Poorer	–	1.05 (1.03–1.08)	<0.001
	Middle	–	0.99 (0.96–1.01)	0.254
	Richer	–	1.04 (1.01–1.07)	0.007
	Richest	–	1.14 (1.10–1.18)	<0.001
Pregnancy intention (v225)	Wanted (Ref)	–	1.00	–
	Wanted later	–	1.25 (1.23–1.28)	<0.001
	Not wanted at all	–	0.54 (0.53–0.56)	<0.001
Employment status (v714)	Not working (Ref)	–	1.00	–
	Working	–	0.90 (0.89–0.92)	<0.001
Media exposure	No exposure (Ref)	–	1.00	–
	Exposed	–	0.97 (0.95–0.99)	0.001
Age group (v013)	15–19 (Ref)	–	1.00	–
	20–24	–	1.00 (0.94–1.07)	0.926
	25–29	–	1.00 (0.94–1.07)	0.985
	30–34	–	0.99 (0.92–1.05)	0.682
	35–39	–	1.01 (0.94–1.07)	0.869
	40–44	–	1.02 (0.96–1.09)	0.531
	45–49	–	1.01 (0.95–1.08)	0.771
Marital status (v501)	Never married (Ref)	–	1.00	–
	Married	<0.001	0.67 (0.63–0.71)	<0.001
	Living with partner	<0.001	0.68 (0.64–0.72)	<0.001
	Widowed	<0.001	0.38 (0.33–0.43)	<0.001
	Divorced	<0.001	0.66 (0.60–0.74)	<0.001
	Separated	<0.001	0.63 (0.57–0.68)	<0.001
Place of residence (v025)	Urban (Ref)	–	1.00	–
	Rural	<0.001	1.02 (1.00–1.05)	0.051

### Model comparison

As Table 4 shows, model 0 had the highest cluster variation (ICC = 0.0778) and the largest information criteria (AIC = 390,232.3, BIC = 390,254). Model 1 had the lowest cluster variation (ICC = 0.0485) and lower AIC and BIC values (AIC = 380,384.2, BIC = 380,656.3), while model 2 had an ICC = 0.0705, AIC = 389,700, and BIC = 389,743.5. Overall, model 3 (the full model) was considered the best-fitting model because it had the lowest AIC (380,384.2) and BIC (380,656.3), with the lowest cluster variation (ICC = 0.0485), indicating better explanatory power compared with the other models.

**Table 4:** Comparison of random effects models.

Parameter	Model 0 (empty)	Model 1 (individual)	Model 2 (community)	Model 3 (full)
Log-likelihood	−195,114.13	−190,167.11	−194,845.99	−190,167.11
AIC	390,232.3	380,384.2	389,700	380,384.2
BIC	390,254	380,656.3	389,743.5	380,656.3
ICC	0.0778	0.0485	0.0705	0.0485
Variance	0.2775	0.1676	0.2494	0.1676

## Discussion

This study revealed that the prevalence of complete antenatal care is 19.94%. This figure is lower than that reported in studies conducted in 13 sub-Saharan African countries (53.8%) (19), Ethiopia (52.3%) (20), Nepal (43%) (21), Malaysia (50%) (22), and Zambia (21.8%) (23, 24). Conversely, it is higher than the prevalence reported in studies conducted in 10 sub-Saharan African countries (10.4%) (14), the East African region (11%), and Nigeria (6.7%). Finally, it is comparable to that of studies conducted in the Democratic Republic of Congo (12.6%) (16, 24). This study indicates that the prevalence is considerably lower than the WHO recommendations for antenatal care, which are designed to ensure a positive pregnancy experience. The WHO envisions a scenario where every pregnant woman and newborn receives high-quality care throughout pregnancy, childbirth, and the postnatal period. ANC is a critical component within the continuum of reproductive healthcare, facilitating essential healthcare activities such as health promotion, screening and diagnosis, and disease prevention. Evidence-based practices in ANC, when implemented in a timely and appropriate manner, have been shown to save lives.

Compared with women living in the poorest households, women living in the poorer (aOR = 1.05, 95% CI: 1.03–1.08), richer (aOR = 1.04, 95% CI: 1.01–1.07), and richest (aOR = 1.14, 95% CI: 1.10–1.18) households had slightly higher odds of receiving all four selected ANC components. However, no significant difference was observed among women in the middle wealth category (aOR = 0.99, 95% CI: 0.96–1.01). Although the associations were modest, greater household wealth may reduce transportation and other indirect costs that limit access to ANC services (17), as has been shown in studies conducted in Ghana (25), Nepal (26), and Kenya (27). Women with a higher wealth index are more likely to have the financial resources required to cover healthcare expenses, including transportation and incidental costs. This financial capability often reduces barriers to accessing complete ANC in sub-Saharan African countries. From a social determinants of health perspective, socioeconomic and demographic characteristics influence ANC quality by shaping women’s knowledge, financial capacity, autonomy, exposure to health information, and ability to access healthcare services. Education may improve health literacy and women’s ability to understand and request recommended ANC services, while household wealth may reduce transportation, diagnostic, and other indirect financial barriers. Employment can provide financial independence, although inflexible working conditions and time constraints may limit service use. Media exposure may increase awareness of ANC recommendations, whereas marital status and household headship may influence social support, control over resources, and healthcare decision-making. Maternal age may reflect differences in pregnancy experience, perceived risk, and care-seeking behaviors. Recent multi-country evidence from Sub-Saharan Africa confirms that education, wealth, residence, marital status, autonomy, and access to health information are important determinants of ANC utilization and the receipt of complete ANC (28).

This study revealed that women with higher educational attainment were significantly more likely to receive all components of ANC compared to those without formal education. Specifically, women with primary education exhibited 63% higher odds of obtaining complete ANC than their counterparts with no formal education. This likelihood increased markedly for women with secondary education, who demonstrated 131% higher odds of receiving comprehensive ANC compared to women lacking formal education. Additionally, women with higher education had 199% higher odds of accessing complete ANC than those without education. These findings suggest that as women’s educational levels rise, their probability of accessing and utilizing comprehensive quality antenatal care services improves significantly. This finding suggests that higher educational attainment positively influences maternal healthcare utilization and was consistent with findings reported in Nigeria (29), Ethiopia (30), Nepal (26), Tanzania (31), and Benin (32). In sub-Saharan countries, women with higher educational attainment are more likely to possess advanced healthcare knowledge, preventive awareness, and improved decision-making capabilities as mothers. Such women are more inclined to participate in ANC due to their heightened awareness of the benefits associated with regular ANC, including reduced pregnancy risks and safer childbirth.

Women who desired a later pregnancy exhibited 1.25 times higher odds of receiving all components of antenatal care, whereas those who did not want the pregnancy had 45% lower odds (aOR = 0.55) compared to those who wanted the pregnancy. This finding aligns with studies conducted in Ethiopia (37) and Guinea (38). Women with intended pregnancies are more likely to complete the recommended ANC visits compared with those whose pregnancies were unintended. This association may reflect greater readiness for pregnancy and motherhood, particularly among adult women. Moreover, pregnancy intention may strengthen women’s health-seeking behavior and decision-making autonomy, leading to increased awareness of the benefits of comprehensive ANC utilization and improved adherence to recommended maternal healthcare services.

All marital status categories exhibited significantly lower odds of receiving complete antenatal care compared to never-married women. The lowest odds were observed among widowed women (aOR = 0.38), followed by separated (aOR = 0.61), divorced (aOR = 0.65), married (aOR = 0.69), and those living with a partner (aOR = 0.68), consistent with previous studies conducted in Ethiopia (33). This pattern may be attributed to various social and behavioral factors. Women who have never married might be younger, possess higher educational attainment, or exhibit greater autonomy in accessing health services. In contrast, women who are married or have been previously married may face household responsibilities, sociocultural constraints, or economic barriers that impede their access to comprehensive ANC. Widowed women, in particular, may experience social isolation or reduced support from partners and families, further decreasing their likelihood of receiving complete ANC. This observation aligns with previous findings that have reported similar associations between marital status and lower uptake of maternal health services.

Media exposure was associated with a slight reduction in the odds of receiving all four selected ANC components (aOR = 0.97, 95% CI: 0.95–0.99). This finding contrasts with previous evidence indicating that exposure to radio and television increases ANC utilization in Sub-Saharan Africa. However, the observed effect represents only a 3% reduction in the odds and, despite its statistical significance in this large sample, may have limited practical importance. Media exposure may improve women’s awareness and encourage ANC attendance, but receiving all four components also depends on facility readiness, availability of supplies, provider practices, and health-system capacity. Moreover, the binary media-exposure measure did not capture the frequency, type, timing, or maternal-health content of the information received. Therefore, this unexpected association may reflect measurement limitations, residual confounding, or unmeasured differences across countries rather than a direct negative effect of media exposure, and it should not be interpreted causally, as this contrasts with findings from Bangladesh (34), Uganda (35), Nepal (26), and Rwanda (36). Maternal exposure to media correlates with heightened awareness of pregnancy-related complications and underscores the importance of optimal use of ANC services. Furthermore, media information may positively affect both the frequency and content of ANC service utilization.

Women living in female-headed households had slightly lower odds of receiving all four selected ANC components than those in male-headed households (aOR = 0.96, 95% CI: 0.94–0.98). This represents only a 4% reduction in the odds and, although statistically significant in this large sample, may have limited practical significance. Female household headship may increase women’s autonomy and control over healthcare decisions; however, it may also indicate widowhood, separation, male-partner migration, limited financial resources, reduced social support, or greater caregiving and household responsibilities. Moreover, the DHS household-headship variable does not directly measure women’s decision-making autonomy or household support. The small association should therefore be interpreted cautiously and may reflect residual socioeconomic differences rather than a direct effect of female household headship. This observation contrasts with findings from West Africa (39) and Sierra Leone (40). The rationale is that female-headed households may possess greater autonomy in accessing healthcare facilities, thereby enhancing the probability of obtaining comprehensive antenatal care services. Concerning employment status, employed women demonstrated slightly lower odds of receiving quality antenatal care compared to their unemployed counterparts (aOR = 0.90, *p* < 0.01). This result differs from a study conducted in Sub-Saharan Africa (27). It is plausible that employed women, who frequently act as the primary economic providers for their families, encounter diminished opportunities to complete antenatal care visits. Although employment can increase women’s financial independence, much female employment in Sub-Saharan Africa is informal, physically demanding, and accompanied by inflexible working hours, limited maternity protection, and no paid leave. Employed women may therefore experience time constraints, loss of earnings, or difficulty obtaining permission to attend and complete ANC services. Moreover, the DHS employment indicator does not distinguish occupation type, working conditions, working hours, job security, or access to maternity benefits. The observed association may consequently reflect precarious employment, residual confounding, or other unmeasured socioeconomic circumstances rather than a direct adverse effect of employment, and it should not be interpreted causally.

## Conclusion

The study indicates that fewer than 20% of women in Sub-Saharan Africa receive all recommended components of antenatal care, highlighting persistently low coverage. The primary determinants of complete antenatal care include household wealth, maternal education, pregnancy intention, marital status, media exposure, sex of the household head, and maternal employment status. These findings emphasize ongoing socioeconomic and demographic inequalities in access to quality maternal health services. Addressing these disparities necessitates targeted interventions, such as promoting female education, expanding health communication through mass media, supporting economically disadvantaged households, and encouraging the active involvement of both men and women in maternal health decision-making. Strengthening policies and programs to ensure equitable access to comprehensive antenatal care, particularly for vulnerable populations, is crucial for improving maternal and neonatal health outcomes and advancing progress toward Sustainable Development Goal 3 in the region.

### Strength and limitations

This study employed DHS data, which are distinguished by their larger sample size and superior quality, thereby significantly reducing the risk of sampling and measurement biases. However, the cross-sectional study design precluded the assessment of causal effects, leaving it uncertain whether the data are time-dependent. A further limitation was the lack of information in the DHS regarding the distance to healthcare facilities and the quality of healthcare providers, both of which could influence the use of ANC services. Additionally, the outcome variable was constructed using four DHS-reported antenatal care components: blood pressure measurement, urine testing, blood testing, and iron supplementation. While these indicators are commonly used to evaluate the content of antenatal care services, they do not encompass all dimensions of antenatal care quality. Critical aspects such as counselling services, continuity of care, provider competence, interpersonal communication, respectful maternity care, client satisfaction, and women’s experiences of care were not available in the DHS dataset. Consequently, the findings should be interpreted as reflecting the receipt of selected essential antenatal care components rather than a comprehensive assessment of overall antenatal care quality. Finally, some potentially significant factors, including distance to health facilities, transportation barriers, health facility readiness, and provider characteristics, were not available in the dataset and could not be examined.

## Data Availability

The raw data supporting the conclusions of this article will be made available by the authors, without undue reservation.
